# Summer rain and wet soil rather than management affect the distribution of a toxic plant in production grasslands

**DOI:** 10.1038/s41598-023-40646-z

**Published:** 2023-08-19

**Authors:** Thomas C. Wagner, Michael Laumer, Gisbert Kuhn, Franziska Mayer, Klaus Gehring, Marie-Therese Krieger, Johannes Kollmann, Harald Albrecht

**Affiliations:** 1 Restoration Ecology, Department of Life Science Systems, TUM School of Life Sciences, Technical University of Munich, Emil-Ramann-Str. 6, 85354 Freising, Germany; 2 Institute for Agroecology and Organic Farming, Bavarian State Research Center for Agriculture (LfL), Lange Point 12, 85354 Freising, Germany; 3 Institute for Plant Protection, Bavarian State Research Center for Agriculture (LfL), Lange Point 10, 85354 Freising, Germany

**Keywords:** Agroecology, Climate-change ecology, Grassland ecology

## Abstract

In the northern forelands of the Alps, farmers report an increase of *Jacobaea aquatica* in production grasslands. Due to its toxicity, the species affects grassland productivity and calls for costly control measures. We are investigating the extent to which management practices or climatic factors are responsible for the increase of the species and how the situation will change due to climate change. We tested for effects of management intensity, fertilization, agri-environmental measures, and soil disturbance, and modeled the occurrence of the species under rcp4.5 and rcp8.5 scenarios. The main determinants of the occurrence of the species are soil type and summer rainfall. A high risk is associated with wet soils and > 400 mm of rain between June and August; an influence of the management-related factors could not be detected. Under the climate-change scenarios, the overall distribution decreases and shifts to the wetter alpine regions. Thus, the current increase is rather a shift in the occurrence of the species due to the altered precipitation situation. Under future climatic conditions, the species will decline and retreat to higher regions in the Alps. This will decrease the risk of forage contamination for production grassland in the lowlands.

## Introduction

Over the past decades, managed grasslands worldwide have become subject to substantial changes. Modified land use, including intensification of current farming, abandonment, afforestation, and conversion to arable land, are the driving forces behind this development^1–4^. While abandonment and afforestation induce a gradual suppression of typical grassland species by tall herbs, shrubs, and trees, intensification usually favors plants adapted to high nutrient availability and frequent cutting. This land-use change results in an overall change in species composition and reduced biodiversity, including the loss of specialists for nutrient-poor soils and the spread of undesired or even poisonous species^3,5–7^. Global warming and altered rainfall patterns significantly reinforce and accelerate this negative development^8^.

Wet grasslands are particularly affected by these changes^8,9^, since more intensive grazing or mowing is often supported by drainage. Thus, specialist species of wet grasslands are faced with two problems, i.e., reduced soil moisture and increased competition^6^. For agricultural usage, such changes of the grasslands can be problematic if the increasing species have poor nutritional value. Examples of such ‘native invaders’ are the poisonous meadow saffron (*Colchicum* sp*.*) or ragwort species (*Senecio* and *Jacobaea* sp.), which contain cytotoxic colchicine or hepatotoxic pyrrolizidine alkaloids, respectively^10–13^. As these toxic substances can cause severe health problems to livestock and humans, they can question further forage usage of the grasslands^10,14^.

A species for which farmers report such a noticeable change in abundance over the past decades is marsh ragwort, *Jacobaea aquatica* (Hill) G. Gaertn et al., a characteristic plant of wet grasslands endemic to Central and Western Europe. Its distribution range in Germany extends from the coastal lowlands to montane grasslands^15^, covering large gradients of precipitation and temperature. In Southern Germany, *J. aquatica* occurs in many regions, while populations in warmer areas with lower rainfall are mainly restricted to moist soils^16^. Furthermore, grassland management seems to have a decisive influence on local abundance. Thus, frequent cutting or mowing during seed set can boost the spread of *J. aquatica*^17,18^. In addition, trampling by livestock or ruts caused by tractors and machinery lead to gaps in the vegetation that provide beneficial microsites for the establishment of this wind-dispersed and short-lived species^17,19,20^. As a consequence, various studies focused on the development of methods to effectively control this poisonous plant (e.g.^21,22^). They recommended avoiding vegetation gaps caused by heavy machinery or livestock trampling on water-saturated soils since dense vegetation prevents seedling establishment^17^. Control of *J. aquatica* can also be achieved by cutting just before the ripening of flower heads^23,24^, by increased shading of a denser canopy due to reduced mowing^22^, by manually removing ragwort plants and roots^25^, or by application of herbicides^18,25,26^. As populations can survive such treatments in the soil seed bank^24,27^, none of these measures result in the complete eradication of *J. aquatica* populations.

For farmers, *J. aquatica* is highly problematic because all plant parts contain high concentrations of pyrrolizidine alkaloids (PA) and PA-N-oxides. Thus, abundant infestation by *J. aquatica* was reported to be the reason why 6% of farmers lost livestock on the Orkney Islands^10^. Contrasting trends were observed in large parts of Central Europe over the past decades: Whereas *J. aquatica* strongly declined in Northern Germany^6,28^, increasing infestations were reported from the northern pre-alpine grasslands of Switzerland, Germany, and Austria^16,17,23^. This contradicting development of the species was also observed in the lowlands and the alpine forelands of Bavaria^16^. However, the mechanisms behind these trends are largely unknown.

Since the agricultural use of lowland grasslands and grasslands in the pre-alpine regions are almost similar, also differences in climate and soil conditions come into question as a cause for this opposite development. By modifying soil moisture, temperature, humidity, and CO_2_ concentrations, climate change can significantly impact both species and functional diversity of wet grasslands^8,29^. Thus, drought-induced by global warming may affect *J. aquatica* either directly or indirectly, e.g., by changing water supply and competition or by interacting with farming activities which create gaps that are suitable for colonization by (non-)native invasive species in grasslands^8^.

Despite the good knowledge of the control of *J. aquatica*, the drivers of the supra-regional changes in the abundance of *J. aquatica* during the past decades are poorly understood. Furthermore, little is known about how management regimes like grazing or mowing, the management intensity, or management systems like organic farming and agri-environmental schemes impact the distribution of *J. aquatica*. Therefore, the objective of our study was to answer the following research questions:

- What are the climatic, soil, and management factors that determine the regional distribution and abundance of *J. aquatica*?
- How is climate change over the past decades related to regionally declining or increasing occurrence of the poisonous species?
- How will future climate change affect the distribution of the species?

To answer these questions, we applied habitat suitability and a random forest model that disentangled the role of soil, climate, and management in the spread of *J. aquatica,* and assessed the climate-related changes in the probability of occurrence over the past decades and the future.

## Methods

### Study region

**Figure 1:**
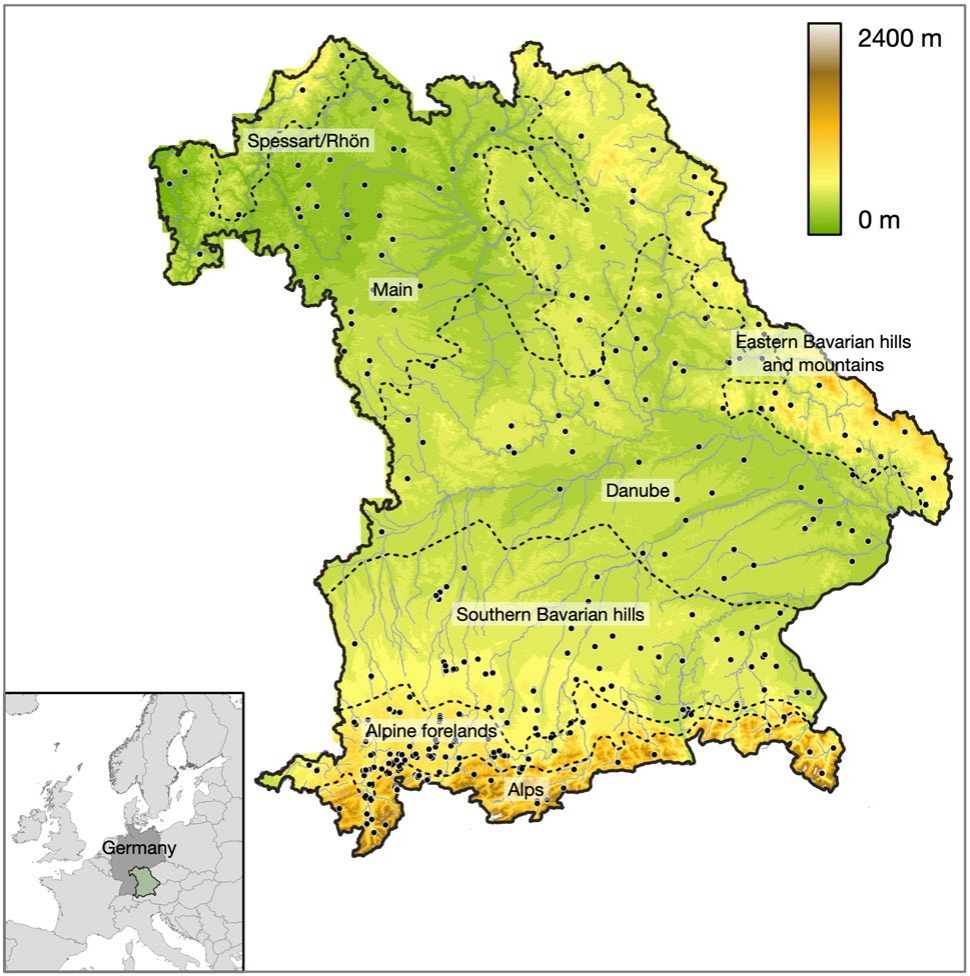
Location of the study sites in Bavaria with seven climate regions separated by dashed black lines (see main text). Black dots indicate sampling sites with *Jacobaea aquatica* and a set of randomly selected pseudo-absences.

Our study covers the state of Bavaria (Germany), which has an area of 70,550 km^2^ (Fig. 1) and is a suitable model region. Bavaria includes seven climatic sub-regions, i.e., the Alps, Alpine Forelands, Southern Bavarian Hills, the Danube and Main regions, the Spessart-Rhön Highlands, and the Eastern Bavarian Hills and Mountains^30^. Mean annual temperatures vary from 8.5 °C in the Main region over 7.3 °C in the Alpine forelands to 5.7 °C in the Alps, with an overall average of 7.9 °C. The warmest months are July and August, with mean temperatures of 16.3 °C; the coldest months are January and February, with − 0.5 °C. Mean temperatures in spring (April–May) reach already 7.7 °C, and the vegetation period lasts from May to October. Precipitation increases with altitude and proximity to the Alps. While the Main region only receives about 700 mm, the alpine forelands get 1500 mm, locally > 2000 mm in the Alps. Precipitation is highest in summer, with 1.5-fold the amount of the other seasons.

In the study region, 10,573 km^2^ is permanent grassland; most (98%) are meadows, mowing pastures, or pastures, while the remaining 2% are litter meadows or conservation grassland^31^. The proportion of grassland varies among regions, ranging from 10 to 30% in the hilly parts of southern Bavaria, the Danube and Main region, and in the Spessart-Rhön region, up to 80–100% in the Alps and their foothills with highest densities in the Oberallgäu and Garmisch-Partenkirchen districts. In the East Bavarian Hills and Mountains, there is a gradient from 30% in the northern to 80% in the southern part of the region. Higher percentages of grassland are usually associated with increased precipitation and a more pronounced relief.

### Study species

*Jacobaea aquatica* (syn. *Senecio aquaticus*; Asteraceae) is native to Western and Central Europe, where it occurs in (semi)natural wet grasslands, grassy floodplains, and along the banks of watercourses^17,32^. The species has a high light demand and is usually biennial. Frequent cutting can prevent flowering and significantly extend its life span^17^. In highly infested grasslands, *J. aquatica* can locally exceed 100 plants per m^2^ (up to 30–50% cover), and each plant may produce several hundred seeds with a pappus facilitating wind dispersal^25^. Seeds germinate quickly under favorable moisture and light conditions^19,22^, and the species forms a persistent soil seed bank of between 350 and 2000 seeds per m^2^^18,25,33^. There is no information on the dispersal distances of *J. aquatica*. However, in the closely related species *J. vulgaris*, 89% of the achenes could not overcome a 5-m distance, and none was found > 14 m^34^. As *J. aquatica* produces considerably larger achenes, long-distance dispersal has apparently even less importance for this species^10^. The high pyrrolizidine content of *J. aquatica* makes the species poisonous for livestock and cattle and can even be harmful to humans via milk^13,35^. Already at a density of one plant per 10 m^2^, grassland is deemed unsuitable for forage purposes for cattle and horses^18^. In Bavaria, *J. aquatica* can be found throughout the entire federal state, while occurrence in warmer regions with lower rainfall is mainly restricted to moist soils^16^.

### Data acquisition and preprocessing

In 2017, 259 fields of 126 farms in the study region were surveyed for the occurrence of *J. aquatica* during the main vegetation period (May–September), with at least 2 weeks between the last cut or grazing. The abundance of *J. aquatica* was determined for each field along two opposing 5-m wide transects, diagonal to the field. As each transect differed in length, this abundance was projected to a standardized plot area with a size of 100 m^2^, and assigned to frequency classes (Supplementary Table A1). Simultaneously, major gaps in the grassland sward, such as tire tracks or livestock tread marks, were recorded.

Additionally, the respective farmers answered a questionnaire including general practices and the specific management of the infested grasslands. It included the type of management (conventional, organic), the implementation of agri-environmental schemes, the frequency and type of grassland use (grazing, mowing, combinations), fertilization (mineral, liquid or stable manure), drainage systems as well as gaps in the sward caused by tires or livestock trampling (Supplementary Table A1). Farm size ranged from 4 to 266 ha, mean farm size was 41 ± 35 ha; 52% of the farms were conventional, and 48% were organic. For 68% of the farms, dairy farming was the main production sector, 12% produced suckler cows, and 20% operated different production types. The average stocking rate was 1.1 livestock units ha^−1^. The main types of grassland used were meadows and hay-grazed meadows (80%), pastures (10%), and litter meadows (10%). 50 reference plots (ca. 20%) were unaffected by *J. aquatica*, the remaining 209 plots were infested with various densities.

To capture the impact of climatic factors and soil type on the general occurrence and changes of *J. aquatica* we used a generalized boosted regression model to predict the species occurrence risk in Bavaria for the periods 1988–1997 (‘past’), 2008–2017 (‘current’), and 2028–2037 (‘future’) under the rcp4.5 and rcp8.5 scenario. The current time period was used for training, and the model was then projected to the past period and the future. For the training of the model, the presence/absence data of *J. aquatica* were complemented by data from the Bavarian survey of habitats with high nature conservation value^36^ sampled from 2008 to 2017. We only used records from quadrants of the habitat survey maps (scale 1:25,000) that were not occupied by our own findings. This survey comprises the species inventory of over 400,000 habitats recorded from 1984 to 2020; however, only 2012 of them were surveyed in the period 2008–2017.

Data collected from 2008 to 2017 were mainly sampled to complete former sampling and to replace outdated records. Therefore, only small areas were mapped in this time period so that the analyzable data are clumped and cover only a small percentage of the study region. Hence only 87 sites from the current period could be used for model training, while 1320 sites, recorded from 1988 to 1997, were used to validate the model prediction for this time period. Thus, 296 presence points for *J. aquatica* could be included. As the absences recorded in our own dataset were placed on the same farms as the presence points, these real absences were excluded from the climate model due to high spatial correlation. Instead, the complete presence data were complemented with ten sets of 296 pseudo-absences each. These pseudo-absences were randomly placed in such quadrants of the habitat survey maps where *J. aquatica* has not been reported until 2018, and with a minimum distance of 3000 m to other growth areas. Each set of pseudo-absences was then combined with our set of presence points to provide the data for ten models.

Considering the preference of *J. aquatica* for wet and water-influenced soils^37^, we further included soil type as a predictor. These data were taken from the digital vector map of Bavaria^38^. This map was compiled from geological and forestry maps (1:25,000), and was validated against soil profiles. For this map, the German soil classification system was used, and therefore, units do not exactly correspond to the World Reference Base for Soil Resources^39^. To improve comprehensibility, the main soil classes were adapted to the international system (Supplementary Table A2). However, as these systems only match at the level of soil classes, German terms were kept for the subclasses. The respective classes and their subclasses were numerically ordered along a gradient of descending soil moisture (Supplementary Table A2). We assumed soil classes to remain constant during the past and future model periods.

The following climate variables were used as predictors for our habitat suitability model: *all-year minimum and maximum air temperature* (2 m AGL)*, mean precipitation per year, in spring* (March–May)*, summer* (June–August) and *autumn* (September–October) as well as the *number of ice, frost,* and *hot days per year*. For Germany, these data were available as interpolated raster data since the 1980s with a resolution of 1 km × 1 km^40^. To relate the recent changes in the distribution of *J. aquatica* in Bavaria to the development of the climate conditions, data from 1988 to 1997 were compared with current records from 2008 to 2017, each sampling period comprising 10 years. For each period, the respective raster data were averaged and confined to the study region.

The future distribution for the decade from 2028 to 2037 was calculated using the climate data predicted under the rcp4.5 and rcp8.5 scenarios by the Bavarian climate model. Data for each year were obtained from the Bavarian Climate Information System^30^ with a resolution of 5 km × 5 km and the respective variables were averaged for the period.

All raster data were projected to ETRS89; EPSG25832 and resampled to a final resolution of 50 m × 50 m to allow for the detailed representation of soil classes.

### Modeling climate and soil effects on plant occurrence

To identify potential habitats for *J. aquatica* based on climate and soil characteristics, we applied generalized boosted regressions using the R packages *gbm*^41^ and *biomod2*^42^ modeling presence/absence of the species with soil type and the averaged climate data for the current period (2008–2017) as predictors (Supplementary Table A3). For tuning purposes, we ran different models with one of the generated presence/absence data sets and an initial parameter set (Supplementary Table A4), and subsequently gradually modified the parameters *shrinkage, interaction depth*, *minimum objects in node,* and *bag fraction.* Each of the resulting models was evaluated using *gbm.perf,* and the respective root mean square error (rsme) was calculated*.* Finally, the parameter set resulting in the lowest rmse was chosen for our actual model. This model was applied to our ten generated sets of presence/pseudo absence data using a random 70% fraction of the data for training and 30% for model evaluation, assuming a prevalence of 0.5.

The resulting models were evaluated by determining the relative importance of the predictor variables, AUC, and TSS. Extrapolation risk due to projection to areas with non-analogous conditions^43^ was assessed using the mop package^44^. ROC curves were generated using the *performance* function of the *ROCR* package^45^, and sensitivity, specificity, and cut-off values were determined using the *roc* function of the *pROC* package (Version 1.18.0; Robin et al.^46^) with the method *best.* Partial dependence plots were generated for the two most influential variables using the *gbm.plot* function^41^. To assess the independence of the two most influential variables, multi-predictor partial dependence plots were generated for each model and averaged.

Then, each model was used to predict the probability of the occurrence of *J. aquatica* in Bavaria based on the predictors for the current and past period and for the future period under scenarios rcp4.5 and rcp8.5. Model predictions for each period were averaged, the ROC curves were plotted, and the final AUC of the averaged prediction was determined. The importance of the respective predictors was averaged from their relative importance in the respective models. Finally, the prediction performance for the period from 1988 until 1997 was validated against the 1320 sites of the Bavarian habitat survey, including *J. aquatica*. To account for possible inaccuracies in the soil and climatic maps, the validation was repeated with a 250-m buffer around the respective biotope.

Finally, to visualize changes between the study periods and to delineate potential current and future risk areas, these areas were identified where the occurrence or the disappearance of *J. aquatica was* predicted by the model for the first time. Additionally, areas where species occurrence was predicted for both periods but the probability of occurrence decreased or increased by more than 20% were indicated.

### Modeling the management effects on plant occurrence

To determine whether and to what extent farm-specific factors and management practices influence the occurrence (presence/absence) or frequency of *J. aquatica* we used a random forest classification approach^47^. Random forest classifiers provide a high prediction accuracy with factorial data, they are less sensitive to over-parameterization and tolerate collinearity^48^. They further provide information about the importance of the single variables which have been recently applied in various fields of ecology. Using the *randomForest* package (Version 4.6-14^49^) in R^50^, we trained and evaluated ten random forest classifiers for the presence/absence and frequency of *J. aquatica*, each constructed of 1000 decision trees based on land-use data (Supplementary Table A1). For each model, our data were randomly divided into a training set with 130 sites and 129 sites used for model evaluation.

Predictor variables were *organic farming* (true/false), *land-use type* (pasture, meadow, mowing pasture, litter meadow or unused), *use intensity* (number of uses per year), *autumn grazing* (yes/no), *fertilization* (yes/no), *implementation of agri-environmental measures* (yes/no), and *apparent soil disturbance* (vehicle tracks, animal tracks, other). To identify the influence of particular practices, each land-use type and the applied fertilizer types (*fertilizer*: *mineral*, *manure*, *slurry*), were additionally included in the model as Boolean type factor, and *use intensity* was converted into a factor as well (Supplementary Table A1). The performance of the resulting model for absence/presence data was assessed using its ROC and AUC value. The respective model for the frequency of *J. aquatica* was assessed by its confusion matrix and overall error.

## Results

### Current and potential distribution of *Jacobaea aquatica*

The gradient-boosted model based on climate parameters and soil type was well suited to predict the potential distribution of *J. aquatica* for both the current and historic climatic situation. The average AUC value for the ten models (mean ± SD) was 0.96 ± 0.01, and the final averaged model achieved an AUC value of 0.98. The best cut-off based on optimal sensitivity and specificity for the averaged model was determined as 0.35, with a specificity of 98% and a sensitivity of 91% (Supplementary Table A5). Taking all variables into account, the proportion of non-analog cells was ca. 11% for the rcp45 scenario and ca. 30% for the rcp85 scenario. The variables frost days, ice days, and hot days which exclusively caused the deviations, were only of minor importance in the model. The removal of these variables reduced the percentage of non-analogous cells to almost 0% (Supplementary Fig. A2).

Applying the averaged model and the cut-off of 0.35 to the past period and validating the prediction results against the occurrences of *J. aquatica* recorded in the Bavarian habitat survey of the respective time period resulted in a true positive rate of 75% when inaccuracies of the soil map were ignored and even 86% when a 250-m buffer was considered. Among the 1320 habitats with *J. aquatica* surveyed between 1988 and 1997, 331 (ca. 25%) occurred in regions where the model did not predict this species. However, when the uncertainty reflecting potential inaccuracies of the soil map and during surveying were included, the rate of false negatives decreased to 14%, and 1135 of 1320 habitats with *J. aquatica* were correctly predicted. A check of the remaining 14% of the recorded presences, where the model excluded species occurrence, revealed that the habitat survey for all these sites indicates wet soil conditions and wet grassland vegetation. A cross-check with the soil map further showed that all but three of these sites were not reflected by the soil map, probably due to the low resolution and lack of precision of this map.

**Figure 2:**
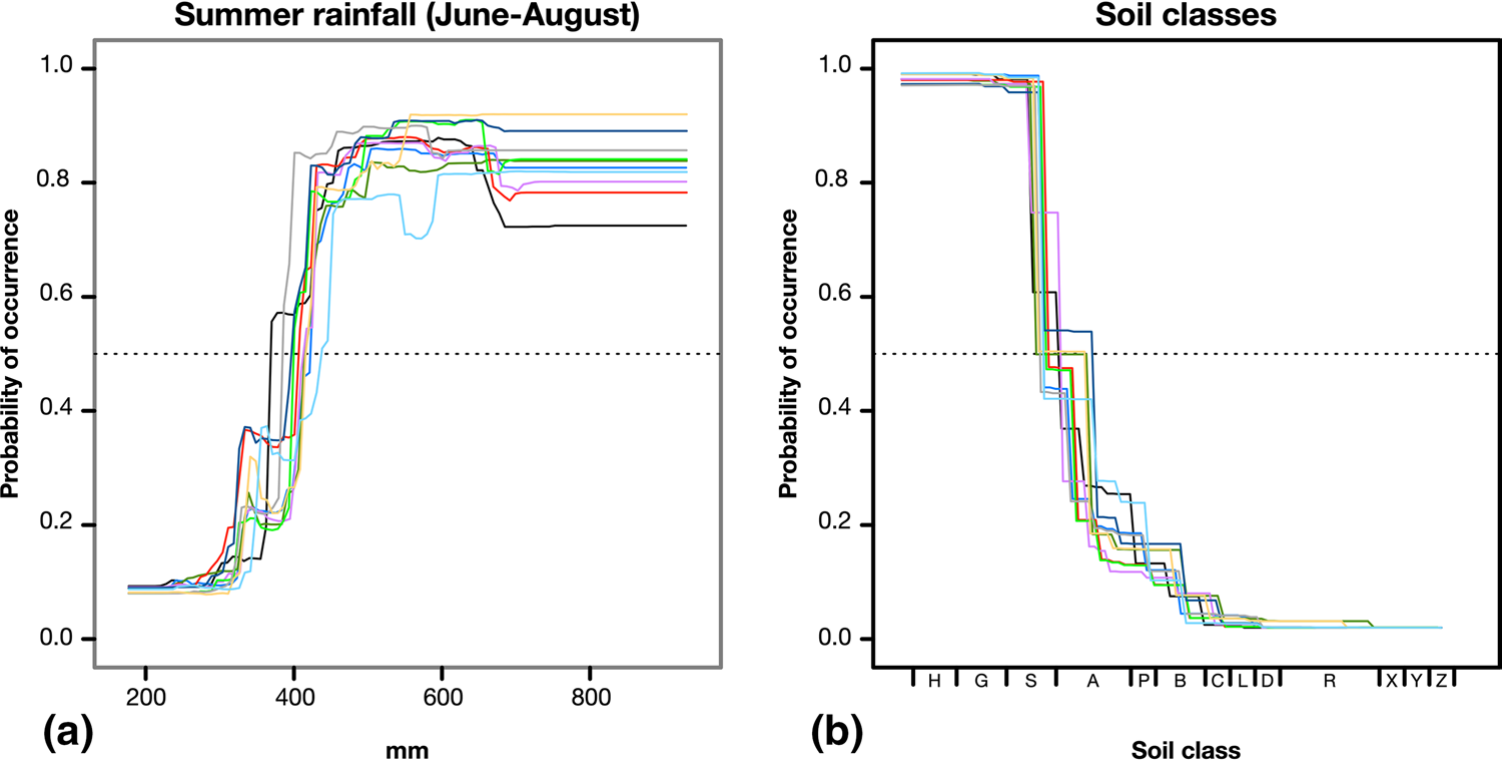
Partial dependence plot of all individual models (in different colors) for the most influential predictors of the occurrence of *Jacobaea aquatica* in Bavarian grasslands: (**a**) summer rainfall (rainJJA), and (**b**) soil class ordered along a gradient of decreasing soil moisture (see Supplementary Table A2).

In all ten individual models, summer rainfall (*rainJJA*; Fig. 2a) was the most important factor for the occurrence of *J. aquatica,* with 49.2 ± 4.5% relative importance, followed by soil type with a relative importance of 15.5 ± 2.3%, and then by the other climatic factors (Supplementary Table A5). The partial dependence plots for summer rainfall reveal a distinctive threshold, i.e., above 406 ± 5 mm of rain in June and August, the probability of *J. aquatica* occurrence raised steeply.

**Figure 3:**
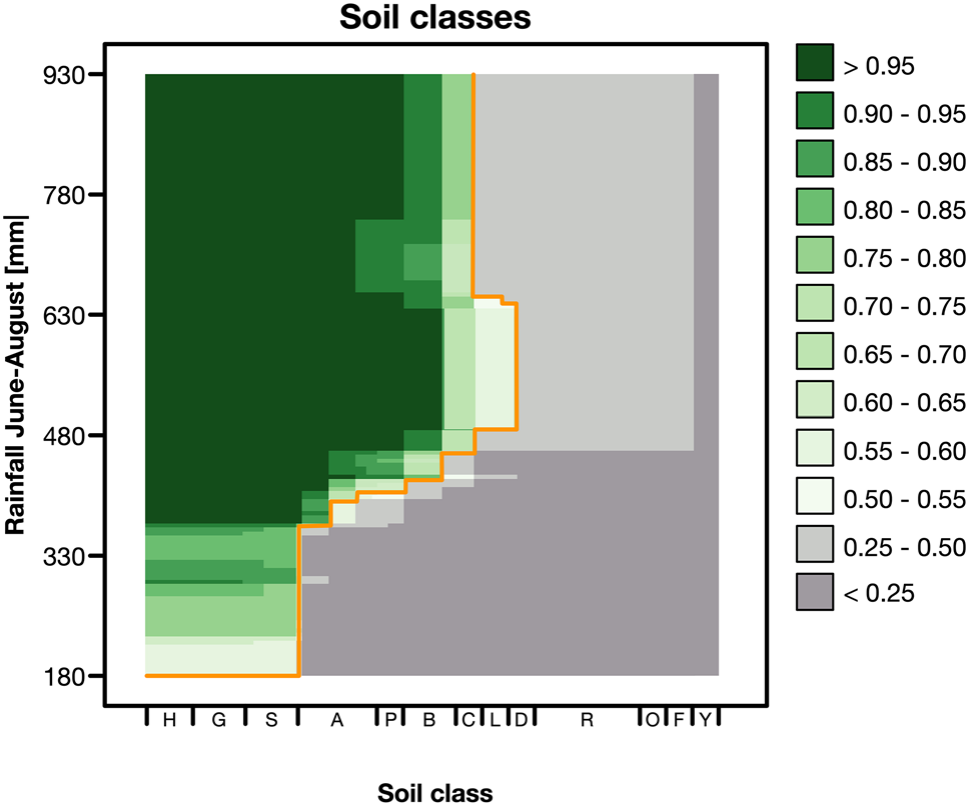
Multi-predictor partial dependence plot with the probability of occurrence of *Jacobaea aquatica* in Bavaria for summer rain (*rainJJA*) and soil type (see Supplementary Table A2: Soil classes). The orange line indicates the cut-off value at 0.35.

A similar but negative threshold was evident for the soil classes (Fig. 2b). The probability of occurrence was restricted to wet soils such as peatlands and water-logged mineral soils, including histosols, gleysols, and stagnosols. In a combined multi-predictor partial dependence plot (Fig. 3) with summer rainfall and soil classes as predictors, however, it became clear that while on these wet soils an occurrence of the species is generally possible, the occurrence on drier soils such as luvisols, pelosols, and cambisols is linked to summer rainfalls > 406 mm. An occurrence on Ah/C soils and terrestrial raw soils can be excluded.

**Figure 4:**
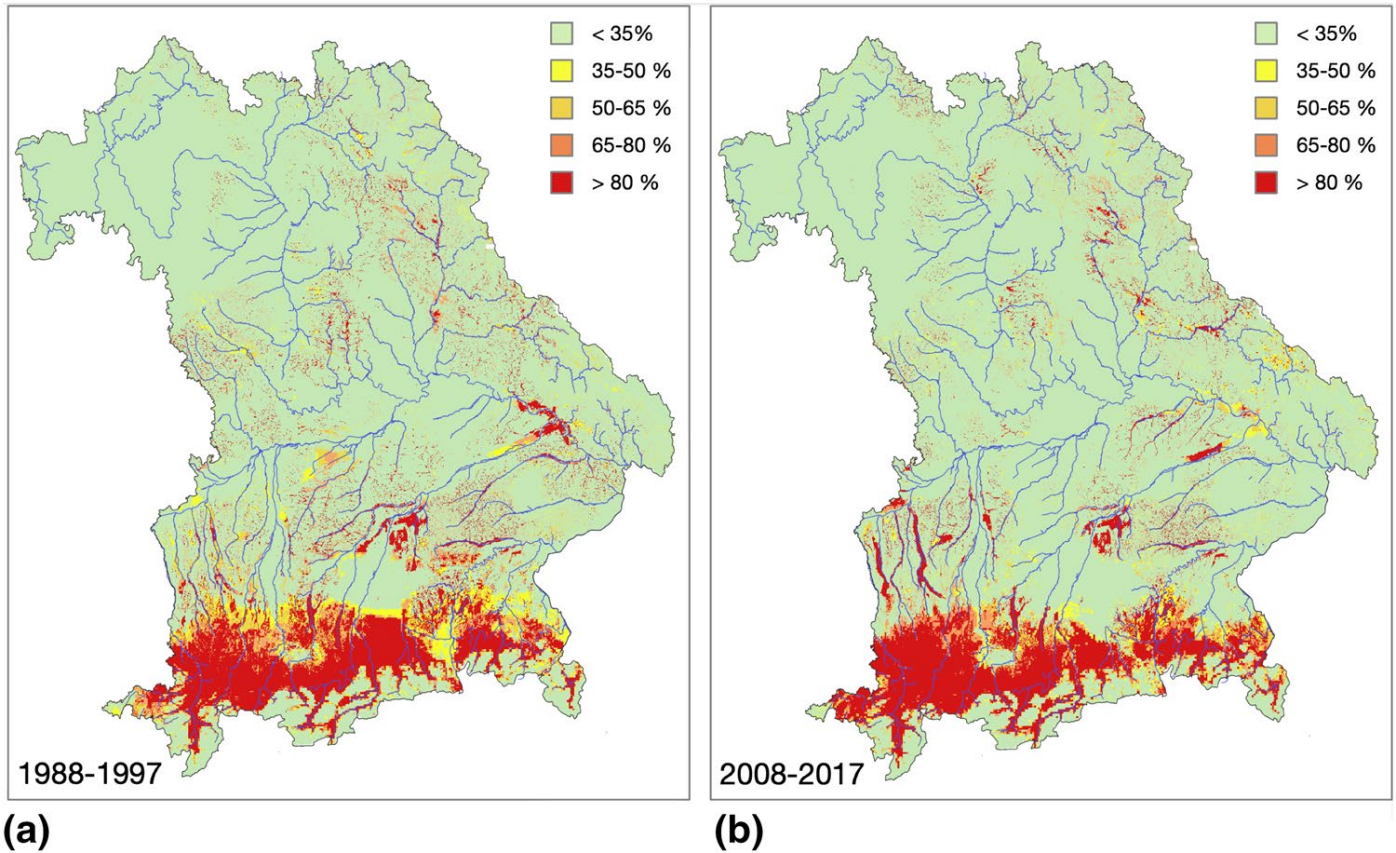
Predicted probability of the potential occurrence of *Jacobaea aquatica* in Bavaria for the time periods 1988*–*1997 and 2008*–*2017 based on an averaged gradient-boosted model. Colors represent percentages of risk areas from green (< 35% of all 50 m × 50 m grid cells show > 60% likelihood of occurrence) to red (> 80% of the cells have a high contamination risk).

For both the past and current periods, the highest risk for infestation and spread of *J. aquatica* was in the climate regions Alpine Forelands and Alps (Fig. 4). There, this risk extended over large and contiguous areas. In the lower and drier regions, probability of occurrence was restricted to floodplains, along streams and rivers, and to peatlands. In the Alps with above-threshold rainfall, the species occurrence was controlled by temperature, namely by the average ice and frost days per year. Thus, the relatively dry Main and Spessart-Rhön regions are low-risk areas.

**Figure 5:**
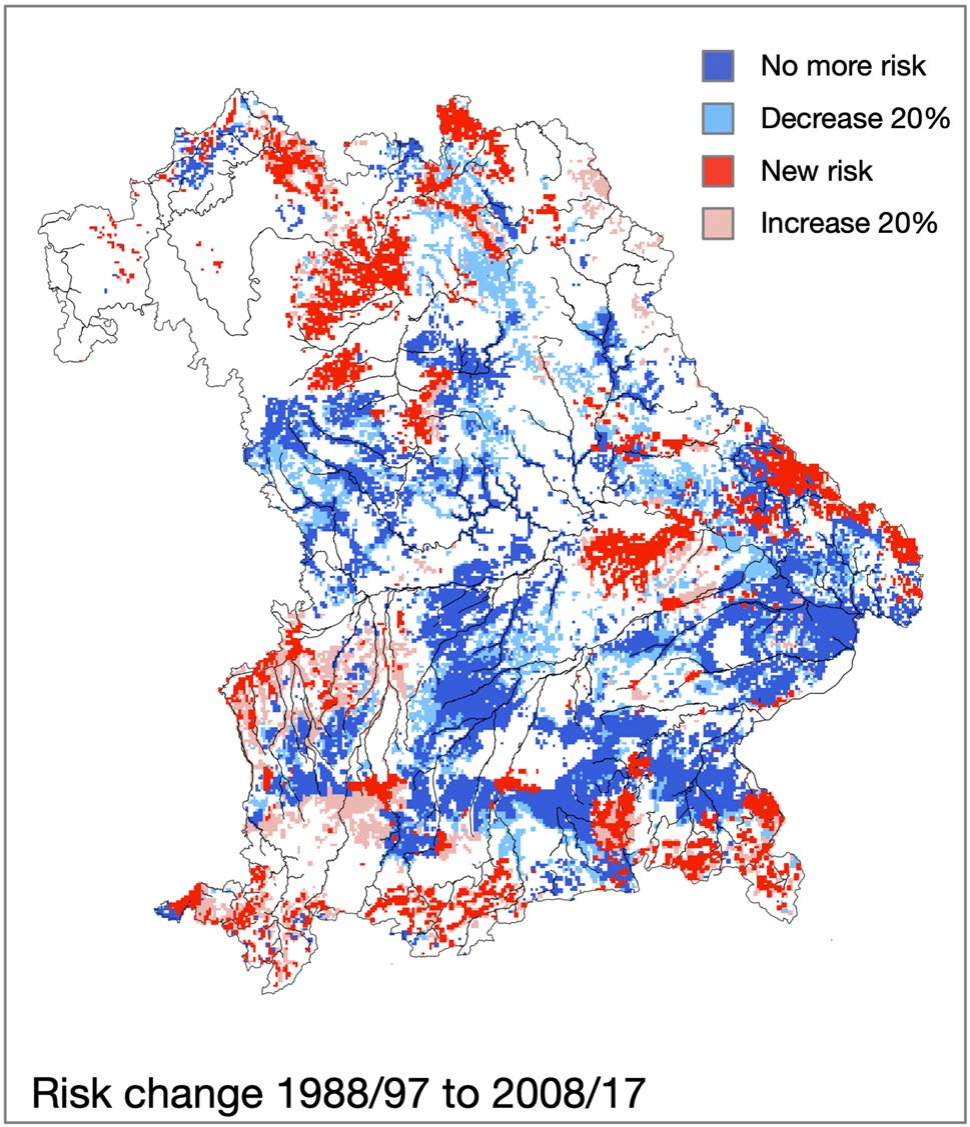
Changes of the occurrence risk of grasslands by *Jacobaea aquatica* in Bavaria from 1988 to 1997 to 2008–2017. Dark red indicates areas where the model predicts a new occurrence of the species in 2007–2017, in the light red areas *J. aquatica* already occurred but the appearance became 20% more likely. Similarly, dark blue indicates areas where the model predicts the extinction of former findings and light blue represents areas where *J. aquatica* already occurred in 1988–1997 but survival became at least 20% more unlikely.

A comparison of the past with the current period clearly shows that the area with an increased infestation risk decreased (Supplementary Table A6). However, this distribution pattern was not uniform across Bavaria. In the Alpine forelands and the Alps, the occurrence probability was still pronounced and the risk extended over a wide contiguous area with even more distinct boundaries. In the rest of the state, occurrences retreated to the wet lowlands along rivers and streams. While *J. aquatica* still occurred in many of these areas in 1988, current conditions made the survival of these populations unlikely, while other regions had shifted from low to high-risk areas (Fig. 5). In total, the risk area decreased by 12% from 15,262 km^2^ in 1988–1997 to 13,452 km^2^ today. An area of 3031 km^2^ where the model predicted a declining risk and 1221 km^2^ where there is an increased risk today contributed to the total change. Of all the areas which exceeded the threshold risk for the occurrence of *J. aquatica* in both time periods, the probability of occurrence had decreased by > 20% on 1372 km^2^, and increased by > 20% on 1438 km^2^ (Fig. 5).

### Predicted distribution of *Jacobaea aquatica*

**Figure 6:**
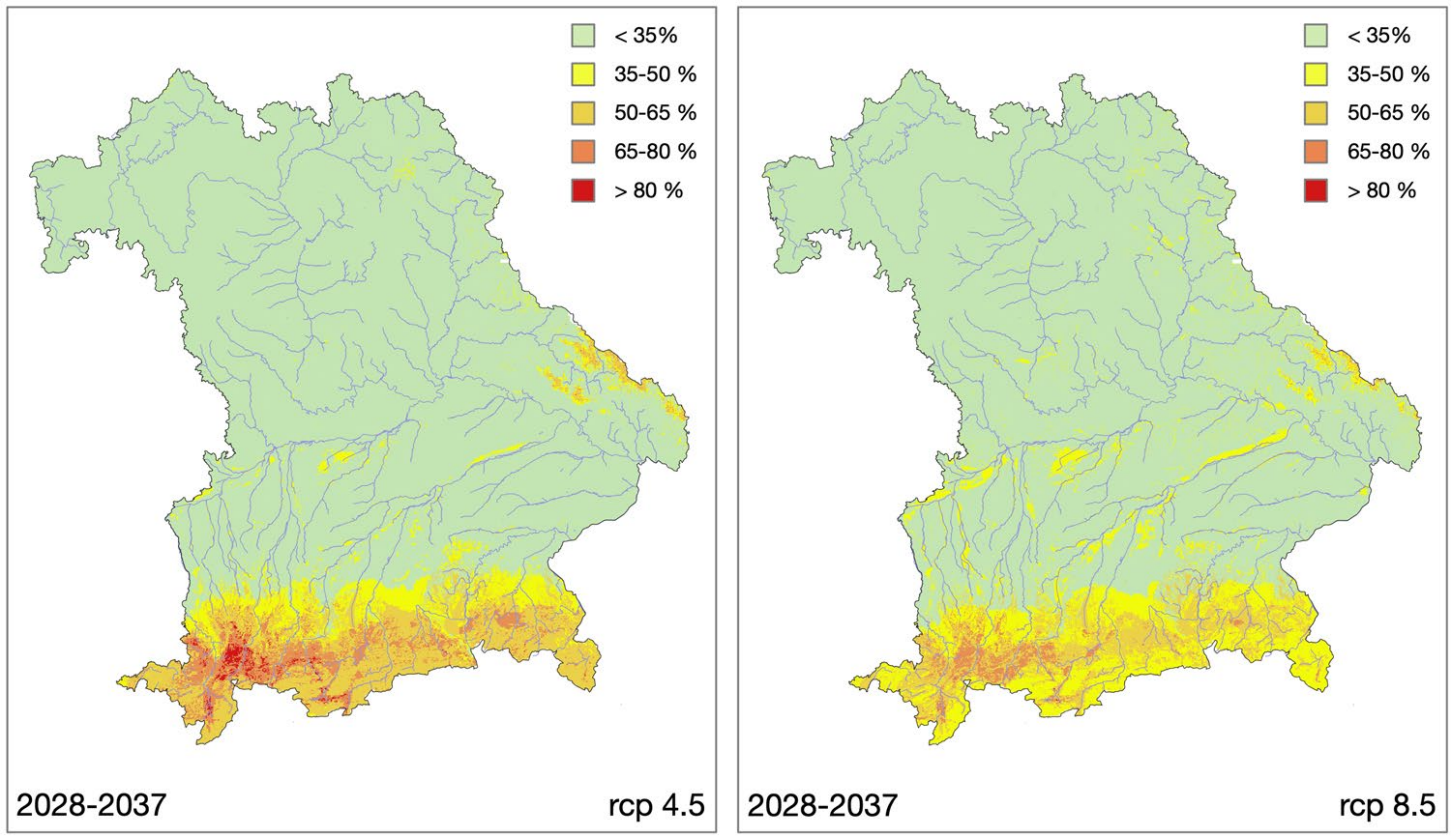
Predicted risk of *Jacobaea aquatica* occurrence in Bavaria for the time period 2028–2037 based on the rcp4.5 and rcp8.5 scenarios, respectively.

The future scenarios both predicted a substantial decrease in risk areas outside the alpine regions of Bavaria (Fig. 6). There was a strong probability that occurrences in the lowlands, which had already decreased in wetlands and river floodplains since 1988, will largely disappear or retreat to larger wetlands and the largest floodplain areas of the Rivers Danube and Isar. This decrease is associated with reduced summer rainfall and mainly affects populations on the wet soil groups of histosols, stagnosols, and gleysols (rcp4.5: 91%; rcp8.5: 82%; Supplementary Table A6). Decreasing summer rainfall also narrowed the extent of the high-risk area in the alpine forelands to areas where summer rainfall remained above the 400 mm threshold or even increased. This risk became more pronounced with scenario rcp8.5, characterized by slightly higher summer precipitation. Both scenarios also predict substantial new risks for higher elevation areas (Supplementary Fig. A1). In addition to future summer rainfall, which will continue to exceed the 406-mm threshold, the decrease of ice and frost days, which limit the occurrence of *J. aquatica* in high precipitation areas, will make this environment accessible.

### Influence of management practice on the occurrence of *Jacobaea aquatica*

**Figure 7:**
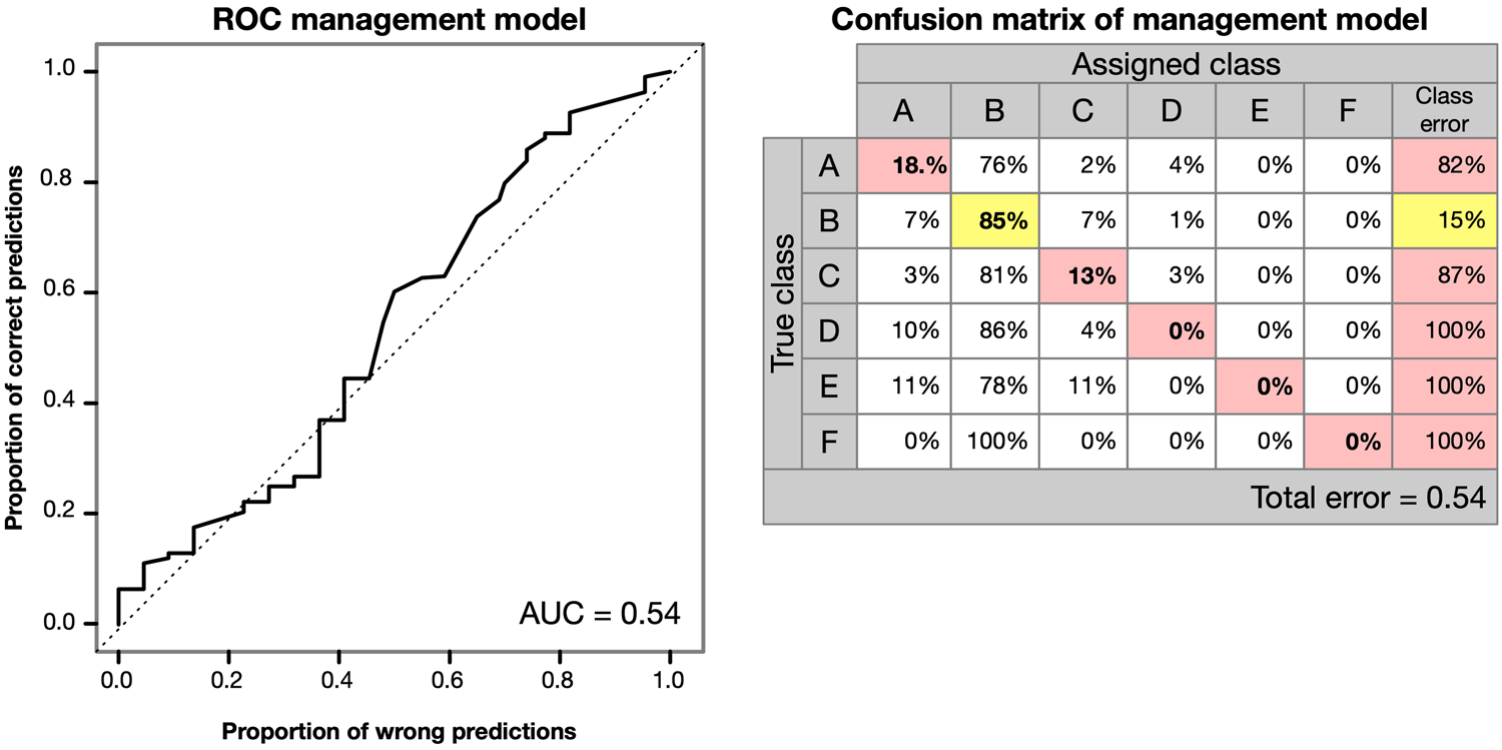
ROC curve of averaged random forest classifiers for the presence of *Jacobaea aquatica* in Bavaria with management parameters as predictors; and confusion matrix of the random forest classifiers for the frequency of the species in dependency of management factors.

In contrast to the clear influence of climate and soil, none of the grassland management variables tested in our models showed a significant impact on the occurrence or frequency of *J. aquatica.* The average AUC value of all ten random forest classifiers for the absence/presence of *J. aquatica* was only 0.469 ± 0.062. Similarly, the random forest model using the species frequency as a response variable had an overall error of 54%, and the confusion matrix shows only a reasonable prediction for the frequency class ‘B’ representing 0–1 individuals/m^2^ (Fig. 7). Hence, as none of the classifiers held any explanatory strength, the tested predictors cannot explain the presence (or absence) or frequency of the species. Consequently, the relative influence of individual variables is not further indicated or discussed.

## Discussion

Our study revealed that the main factors determining the present distribution of *J. aquatica* in Central Europe are abundant summer rainfall and wet soils. With a mean average summer precipitation (June–August) of over 400 mm, *J. aquatica* grows on a broad range of soil types, including *histosols, gleysols, stagnosols, luvisols, pelosols,* and *cambisols*. When the average summer precipitation drops below this threshold, the occurrence of the species becomes increasingly restricted to wet and moist soil types, mainly occurring in peatlands, floodplains, or other wetlands. When summer precipitation falls below 200 mm, the occurrence of *J. aquatica* becomes unlikely. This preference for moist soils agrees with Forbes^10^, who described severe *J. aquatica* infestation problems on the Orkney Islands and found that “soil surface wetness” was the most significant predictor for the occurrence of the target species. The minor influence of precipitation there may be due to climatic differences. Mean annual temperatures 9 K below those in the Alpine foreland and the wide distribution of peat soils^51^ suggest that summer precipitation does not limit the growth of *J. aquatica* on the Orkney islands due to low evapotranspiration. In our study region, temperatures had little influence on the occurrence of *J. aquatica*. Only in the higher elevated areas of the Alps, the number of frost and ice days limit the distribution despite the high summer precipitation.

Modeling the development of species in dependence on environmental trends has become an important tool to understand ecosystem changes and to devise sustainable management strategies^52,53^. Many of these models have been designed to predict the spread of harmful organisms and to limit actual and future damages^54^. Most of these target organisms were invasive species with a negative impact on native ecosystems. *Jacobaea aquatica* can be considered a ‘native invader’^55^, which also shows a considerable spread at least in parts of its distribution range^16,56,57^). For the territory of Bavaria, however, our study could not confirm a significant spread of this problematic species. In contrast, the overall area at risk considerably decreased since 1988–1997, and the probability of occurrence in the affected areas declined. The reason for this is a decrease in summer precipitation, especially in the lower Alpine forelands. A soil type particularly affected by this development is gleysol. Today, the risk areas are mainly restricted to two habitat types: (1) a narrow, sharply delineated area along the northern edge of the Alps where summer rainfall decreased only slightly or even increased, and (2) wet soils of wetlands and riverine lowlands where the previous water regime remained unchanged.

Our models show a clear decrease in the species frequency since the 1990s accompanied by a distinct shift in the regional occurrence mainly due to a change in rainfall distribution. Here, our findings confirm an earlier study by Suttner et al.^16^, who also observed a decline at most of the monitoring points and a shift in the distribution patterns. However, our risk shifts modeled are not fully congruent to the changes observed by Suttner et al.^16^ as these authors only used data from the ‘Bavarian Biotope Mapping Database’, which mainly includes protected areas and not agricultural land. Nevertheless, both analyses come to the conclusion that the ‘increase’ reported by practitioners is more of a local phenomenon and does not represent an overall direction in the spatial distribution of *J. aquatica*.

The trends detected for the recent past will continue in the future: both climate scenarios rcp4.5 and rcp8.5 predict decreasing summer rainfalls and increasing temperatures until 2037. Most likely, decreasing summer rainfall will move the potential risk areas to higher altitudes with sufficient rainfall, where low temperatures actually limit growth. In the lowlands, the risk areas along streams and rivers and the general probability of occurrence will decrease. This effect is even more pronounced with the rcp8.5 scenario, where the expected summer rainfall decrease is higher. As a result of climate change, such shifts and the retreat to higher altitudes are observed or expected for many species^58–60^.

Due to higher rainfalls and temperatures and the considerable decrease of frost and ice days predicted by both climate scenarios, an expansion of *J. aquatica* to higher altitudes in the Alps can be expected creating new potential risk areas. In the rcp8.5 scenario, however, this is mitigated by a stronger decrease in precipitation also in higher regions, while the decrease of risk in the Alpine forelands is somewhat less due to slightly higher precipitation there. However, as these new risk areas are comparatively small, they will not compensate for the areas with reduced risk. For agricultural practice, problems due to the spread of *J. aquatica* to higher altitudes can be estimated as low due to the minor importance of agriculture there. For the riverine grasslands on wet histosols, stagnosols, and gleysols in the extra-Alpine lowlands, our models also predict a decreasing infestation risk which indicates a substantial reduction of future management problems by *J. aquatica* there.

A climate change phenomenon which could not be reflected by our model is the expected increase in the torrentiality of precipitation events in summer^30^. The reason for this is the lack of high-resolution data. However, it can be assumed that more torrential precipitation will impair water infiltration by soils and therefore reinforce soil dryness, further reducing the infestation risk.

Management practice did not show significant effects on the occurrence of *J. aquatica* in our study. Neither organic vs. conventional farming or the implication of conservation schemes, nor land-use intensity in the form of stocking rates, the frequency of mowing and grazing, or the type and amount of fertilizationi or the showed an impact on *J. aquatica* occurrence.

While these results agree well with Forbes^10^ who also found little or no impact of fertilization, cutting frequencies, or stocking rates on the occurrence of *J. aquatica* on 96 farms in Scotland, it contradicts various studies that report significant effects of different management practices from field experiments (e.g.^16,17,21,23,25^). A major reason for this contradiction may be that the management methods applied in the experiments were specifically targeted at reducing *J. aquatica* populations. In contrast, our study and the analysis of Forbes^10^ reflect the actual farming practice where the cost-effective achievement of fodder yields rather determines management decisions than on targeted weed control. Furthermore, *J. aquatica* does not always respond linearly to control measures and complex interactions of different measures also play a role^21,23^. In the study area, *J. aquatica* mainly occurs in wet meadows with a low to intermediate management intensity^21^. At this level of land-use intensity, conventional and organic grassland farming have much of their management practices in common, and the most noteworthy difference between the systems is in the application of organic or mineral fertilizers. As both types of fertilizers similarly stimulate the growth of *J. aquatica*, no significant difference between the effects of the two systems could be detected.

Also, interactions between the schedule of management measures and population development can play a decisive role in establishing the species. Although *J. aquatica* may find favorable site conditions within a risk area, it needs dispersal and suitable germination conditions to colonize and infest potential areas.

Generally, the production of large numbers of wind-dispersed seeds enables *J. aquatica* to rapidly occupy areas in the vicinity of existing populations. Due to the light requirement of establishing seedlings, gaps in the grassland sward essentially facilitate the colonization of so far unoccupied areas^22^. Therefore, maintaining a close and vigorous plant canopy is an important tool to prevent infestation^19^. In our study, however, no such correlation between *J. aquatica* infestation and the occurrence of vegetation gaps was observed. Several reasons could have caused this result. Hence, when phases of seed dispersal and the availability of suitable gaps do not overlap, the risk of seed predation, mortality or false germination is substantially increased. Considering the short dispersal distances of *Jacobaea* sp. seeds^10,19,34^ and the impact of wind direction, gaps suitable for germination may not have been close enough to seed-producing *J. aquatica* plants in our study.

Due to the relevance of the spread of the poisonous plant, *J. aquatica's* spatial and temporal interactions between seed production, seed dispersal, and the accessibility of sites with suitable germination conditions should become an important issue of further experiments and models. For practical farmers, prevention of seed production and maintenance of a close grassland sward should become essential precautionary measures to avoid future infestation problems.

## Conclusions

The occurrence of *Jacobaea aquatica*, an indigenous species of wet grassland in Central Europe, is mainly determined by climatic factors. Although individual, targeted management measures in controlled experiments showed to reduce or avoid infestations by J. aquatica, our study shows that in the real, complex agricultural environment different management does not influence the occurrence of the species. Simple solutions such as extensification or organic farming as a solution to avoid *J. aquatica* infestation are therefore out of the question.

Our study reveals how decreasing precipitation can deteriorate habitat quality mainly on terrestrial soils and thus lead to a large-scale decline of this species. However, this study also displays that the habitat conditions of *J. aquatica* have not deteriorated across its whole distribution range. In some regions where climate change does not negatively affect the living conditions, the species even show an increase.

Our results agree with climate models, which generally indicate that future decline in precipitation patterns will considerably reinforce such losses of species richness in wet grassland. Considering the high nature conservation value and the strong decline of such habitats, measures to preserve these ecosystems by rewetting and extensive management seem appropriate. On the other hand, this decline of highly poisonous *J. aquatica* will also facilitate the production of uncontaminated food for humans and animals. Therefore, a major future challenge for the management of *J. aquatica*  habitats is to find a way to meet both objectives, the conservation of the species threatened by climate and land-use change and the production of healthy food.

### Supplementary Information

Supplementary Information.

## Data Availability

The data that support the findings of this study are available on request from the corresponding author (TCW).

## References

[1] Dallimer M, Tinch D, Acs S, Hanley N, Southall HR, Gaston KJ, Armsworth PR (2009). 100 years of change: Examining agricultural trends, habitat change and stakeholder perceptions through the 20th century. J. Appl. Ecol..

[2] Habel JC, Dengler J, Janišová M, Török P, Wellstein C, Wiezik M (2013). European grassland ecosystems: Threatened hotspots of biodiversity. Biodivers. Conserv..

[3] Koch C, Conradi T, Gossner MM, Hermann JM, Leidinger J, Meyer ST, Overbeck GE, Weisser WW, Kollmann J (2016). Management intensity and temporary conversion to other land-use types affect plant diversity and species composition of subtropical grasslands in southern Brazil. Appl. Veg. Sci..

[4] Schwaiger H, Lenzer B, Essl F (2022). No species loss, but pronounced species turnover in grasslands in the Northern Alps over 25 years. Appl. Veg. Sci..

[5] Catford JA, Jones LP, Gibson DJ, Newman J (2019). Grassland invasion in a changing climate. Grasslands and Climate Change.

[6] Diekmann M, Andres C, Becker T, Bennie J, Blüml V, Bullock JM (2019). Patterns of long-term vegetation change vary between different types of semi-natural grasslands in Western and Central Europe. J. Veg. Sci..

[7] Gibson DJ, Newman JA, Gibson DJ, Newman JA (2019). Grasslands and climate change: An overview. Grasslands and Climate Change.

[8] Joyce CB, Simpson M, Casanova M (2016). Future wet grasslands: Ecological implications of climate change. Ecosyst. Health Sustain..

[9] Lepš J (1999). Nutrient status disturbance and competition: An experimental test of relationships in a wet meadow. J. Veg. Sci..

[10] Forbes JC (1976). Influence of management and environmental factors on the distribution of the marsh ragwort (*Senecio aquaticus* Huds.) in Agricultural Grassland in Orkney. J. Appl. Ecol..

[11] Molyneux RJ, Gardner DL, Colegate SM, Edgar JA (2011). Pyrrolizidine alkaloid toxicity in livestock: A paradigm for human poisoning?. Food Addit. Contam. Part A.

[12] Winter S, Jung LS, Eckstein RL, Otte A, Donath TW, Kriechbaum M (2014). Control of the toxic plant *Colchicum autumnale* in semi-natural grasslands: Effects of cutting treatments on demography and diversity. J. Appl. Ecol..

[13] Gottschalk C, Ostertag J, Meyer K, Gehring K, Thyssen S, Gareis M (2018). Influence of grass pellet production on pyrrolizidine alkaloids occurring in *Senecio aquaticus*-infested grassland. Food Addit. Contam. Part A.

[14] Gottschalk C, Ronczka S, Preiß-Weigert A, Ostertag J, Klaffke H, Schafft M, Lahrssen-Wiederholt M (2015). Pyrrolizidine alkaloids in natural and experimental grass silages and implications for feed safety. Anim. Feed Sci. Technol..

[15] Dörr E, Lippert W (2004). Flora des Allgäus und seiner Umgebung.

[16] Suttner G, Weisser WW, Kollmann J (2016). Hat die Problemart *Senecio aquaticus* (Wasser-Greiskraut) im Grünland zugenommen? Auswertung der Biotopkartierungen 1984–1995 und 1999–2013 in Bayern. Nat. Landschaft.

[17] Suter M, Lüscher A (2008). Occurrence of *Senecio aquaticus* in relation to grassland management. Appl. Veg. Sci..

[18] Gehring, K., Albrecht, H., Kollmann, J., Kuhn, G., Ditton, J., Teixeira, L., Linderl, L., Laumer, M., Mayer, F., Wagner, T., & Gottschalk, C. Effektives Management von Wasser-Kreuzkraut in bayerischem Grünland. Projektbericht. Bayerische Landesanstalt für Landwirtschaft (Ed.): LfL Schriftenreihe, Freising (2021).

[19] Suter M, Lüscher A (2012). Rapid and high seed germination and large soil seed bank of *Senecio aquaticus* in managed grassland. Sci. World J..

[20] Hennings, H. Landschaftsökologische Analyse des Vorkommens von *Senecio aquaticus* (Wasser-Kreuzkraut) in voralpinen Feuchtwiesen. Master Thesis, TU Munich (2013).

[21] Krieger M-T, Ditton J, Albrecht H, Baaij BM, Kollmann J, Teixeira LH (2022). Controlling the abundance of a native invasive plant does not affect species richness or functional diversity of wet grasslands. Appl. Veg. Sci..

[22] Krieger MT, Ditton J, Albrecht H, Linderl L, Kollmann J, Teixeira LH (2022). Effects of shading and site conditions on vegetative and generative growth of a native grassland invader. Ecol. Eng..

[23] Bassler G, Karrer G, Kriechbaum M (2016). The impact of different cutting regimes on population density of *Jacobaea*
*aquatica* (Hill) G. Gaertn., B. Mey. and Scherb. and grassland vegetation. Agric. Ecosyst. Environ..

[24] Albrecht H, Ditton J, Kuhn G, Kollmann J, Krieger MT, Mayer F, Teixeira L, Gehring K (2022). Management von Wasser-Greiskraut (*Jacobaea aquatica*) in Wirtschaftsgrünland des ökologischen Landbaus. Julius-Kühn-Archiv.

[25] Suter M, Lüscher A (2011). Measures for the control of *Senecio aquaticus* in managed grassland. Weed Res..

[26] Gehring K, Thyssen S (2016). Regulierungsmöglichkeiten von Wasser-Kreuzkraut (*Senecio aquaticus*) im Dauergrünland. Julius-Kühn-Archiv.

[27] Suter M, Stutz CM, Gago R, Lüscher A (2012). Lässt sich Wasser-Kreuzkraut in landwirtschaftlichem Grasland kontrollieren?. Agrarforschung Schweiz.

[28] Immoor A, Zacharias D, Müller J, Diekmann M (2017). A re-visitation study (1948–2015) of wet grassland vegetation in the Stedinger Land near Bremen, North-western Germany. Tuexenia.

[29] Brotherton SJ, Joyce CB (2015). Extreme climate events and wet grasslands: Plant traits for ecological resilience. Hydrobiologia.

[30] Bayerisches Landesamt für Umwelt. *Bavarian Climate Information System*. https://klimainformationssystem.bayern.de. Accessed 10 March 2022 (2022).

[31] Bayerisches Landesamt für Statistik (Ed.). *Ernte der Feldfrüchte und des Grünlandes in Bayern 2020*. https://www.statistik.bayern.de/mam/produkte/veroffentlichungen/statistische_berichte/c2103c_202051.pdf (2020).

[32] Sebald O, Seibold S, Philippi G (1999). Die Farn- und Blütenpflanzen Baden-Württembergs.

[33] Bassler G, Karrer G, Kriechbaum M (2013). Mechanical control of marsh ragwort (*Senecio aquaticus* Hill) by cutting. Grassl. Sci. Eur..

[34] McEvoy PB, Cox CS (1987). Wind dispersal distances in dimorphic achenes of ragwort, *Senecio*
*jacobaea*. Ecology.

[35] Kalač P, Kaltner F (2021). Pyrrolizidine alkaloids of European Senecio/Jacobaea species in forage and their carry-over to milk: A review. Anim. Feed Sci. Technol..

[36] Bayerisches Landesamt für Umwelt. *FIS-Natur, Biotopkartierung*. https://www.lfu.bayern.de/natur/biotopkartierung. Accessed 15 March 2021 (2021a).

[37] Bartelheimer M, Gowing D, Silvertown J (2010). Explaining hydrological niches: The decisive role of below-ground competition in two closely related *Senecio* species. J. Ecol..

[38] Bayerisches Landesamt für Umwelt. *Standortliche Bodenkarte von Bayern 1:25.000 und 1:50.000*. https://www.lfu.bayern.de/gdi/wms/boden/uebk25. Accessed 15 March 2021 (2021b).

[39] IUSS Working Group WRB. World Reference Base for Soil Resources. International soil classification system for naming soils and creating legends for soil maps, 4th edn. (International Union of Soil Sciences (IUSS), Vienna, 2022). https://www3.ls.tum.de/fileadmin/w00bds/boku/downloads/wrb/WRB_fourth_edition_2022-12-18.pdf.

[40] Deutscher Wetterdienst. *DWD Open Data*. opendata.dwd.de. Accessed 15 March 2022 (2022).

[41] Greenwell B., Boehmke B., Cunningham J., & GBM Developers. *gbm: Generalized Boosted Regression Models*. R package version 2.1.8. https://CRAN.R-project.org/package=gbm (2020).

[42] Thuiller, W., Georges, D., Engler, R., & Breiner F. *biomod2: Ensemble Platform for Species Distribution Modeling*. R package version 3.4.6. https://CRAN.R-project.org/package=biomod2 (2021).

[43] Owens HL, Campbell LP, Dornak LL, Saupe EE, Barve N, Soberón J, Ingenloff K, Lira-Noriega A, Hensz CM, Myers CE, Peterson AT (2013). Constraints on interpretation of ecological niche models by limited environmental ranges on calibration areas. Ecol. Model..

[44] Cobos, M., Owens, H., Soberón, J. & Peterson, A. *mop: Mobility Oriented-Parity Metric*. R package version 0.1.1. https://CRAN.R-project.org/package=mop (2023).

[45] Sing T, Sander O, Beerenwinkel N, Lengauer T (2005). ROCR: Visualizing classifier performance in R. Bioinformatics.

[46] Robin X, Turck N, Hainard A, Tiberti N, Lisacek F, Sanchez JC, Müller M (2011). pROC: An open-source package for R and S+ to analyze and compare ROC curves. BMC Bioinform..

[47] Breiman L (2001). Random forests. Mach. Learn..

[48] Tagliamonte SA, Baayen RH (2012). Models, forests, and trees of York English: Was/were variation as a case study for statistical practice. Lang. Var. Change.

[49] Liaw A, Wiener M (2002). Classification and regression by randomForest. R News.

[50] R Core Team. *R: A Language and Environment for Statistical Computing* (R Foundation for Statistical Computing, Vienna, 2020). https://www.R-project.org/.

[51] | Scottish Environment Protection Agency. *National Soil Map of Scotland*. https://soils.environment.gov.scot/maps/soil-maps/national-soil-map-of-scotland/ (2022)

[52] Pompe S, Hanspach J, Badeck F, Klotz S, Thuiller W, Kühn I (2008). Climate and land use change impacts on plant distributions in Germany. Biol. Lett..

[53] Thuiller W, Albert C, Aranjo MB, Berry PM, Cabeza M, Guisan A, Hickler T, Midgely GF, Paterson J, Schurr FN, Sykes MT, Zimmermann NE (2008). Predicting global change impacts on plant species distributions: Future challenges. Perspect. Plant Ecol. Evol. Syst..

[54] Runquist RD, Lake T, Tiffin P, Moeller DA (2019). Species distribution models throughout the invasion history of *Palmer*
*amaranth* predict regions at risk of future invasion and reveal challenges with modeling rapidly shifting geographic ranges. Sci. Rep..

[55] Valéry L, Fritz H, Lefeuvre JC, Simberloff D (2009). Invasive species can also be native. Trends Ecol. Evol..

[56] Bosshard A, Joshi J, Lüscher A, Schaffner U (2003). Jakobs- und andere Kreuzkraut-Arten: Eine Standortbestimmung. Agrarforschung.

[57] Liehl M, Bassler G, Kriechbaum M (2012). Das Wasser-Greiskraut (*Senecio aquaticus*) im Bezirk Gmünd, Niederösterreich—Verbreitung, Standortpräferenz und Bewirtschaftungseinflüsse. Mitt. Niederösterr. Landesmus..

[58] Engler R, Randin CF, Thuiller W, Dullinger S, Zimmermann NE, Araujo MB, Pearman PB, Le Lay G, Piedallu C, Albert CH, Choler P, Guisan A (2011). 21st century climate change threatens mountain flora unequally across Europe. Glob. Change Biol..

[59] Vitasse Y, Ursenbacher S, Klein G, Bohnenstengel T, Chittaro Y, Delestrade A, Monnerat C, Rebetez M, Rixen C, Strebel N, Schmidt BR, Lenoir J (2021). Phenological and elevational shifts of plants, animals and fungi under climate change in the European Alps. Biol. Rev..

[60] Iseli E, Chisholm C, Lenoir J, Haider S, Seipel T, Barros A, Hargreaves AL, Kardol P, Lembrechts JJ, McDougall K, Rashid I, Alexander JM (2023). Rapid upwards spread of non-native plants in mountains across continents. Nat. Ecol. Evol..

