# Supplementary material for: Summer rain and wet soil rather than management affect the distribution of a toxic plant in production grasslands
Source: Sci Rep. 2023 Aug 19;13:13530. doi: 10.1038/s41598-023-40646-z (PMC10439881; doi:10.1038/s41598-023-40646-z)
Supplement: Supplementary file 1 — Supplementary Information. [file 41598_2023_40646_MOESM1_ESM.pdf]

Summer rain and wet soil rather than management affect the distribution of a toxic plant in production grasslands

Wagner, Thomas C; Laumer, Michael; Kuhn, Gisbert; Mayer, Franziska; Gehring, Klaus; Krieger, Marie-Therese; Kollmann, Johannes; Albrecht, Harald

Appendix

Table A1: Parameters included in the management model on the distribution of *Jacobaea aquatica* in Bavarian grasslands.

**Factor**	**Factor levels**	**Description**
J. aquatica Absence/presence	0/1	Absence/presence of *Jacobaea aquatica*
J. aquatica Frequency class	A:0,<br>B: 0-1,<br>C: 1-10,<br>D: 10-100,<br>E: 100-1000,<br>F: >1000	Estimated frequency of *Jacobaea aquatica* in individuals/100-m2
Organic Farming	True/False	
Land use type (LUT)		Predominant use of the site
- Pasture	True/false	
- Meadow	True/false	
- Mowing pasture	True/false	
- Litter meadow	True/false	
- Unused	True/false	
Use intensity	1 to 6	Number of cutting or grazing events per year; as factor
Grazing in autumn	True/False	Post-grazing after hey production in autumn
Fertilizer (FER)	True/False	Application of Fertilizer
- FER Mineral	True/False	Application of mineral fertilizer
- FER Manure	True/False	Application of manure
- FER Slurry	True/False	Application of slurry
Agri-environmental measures (AUM)	True/False	Agri-environmental schemes implemented on the field
Disturbance (DIS)	True/False	Apparent soil disturbance, such as vehicle tracks, animal tracks etc.

Table A2: List of soil classes (IUSS Working Group WRB, 2022) and subclasses recorded in the Bavarian soil map (Landesamt für Umwelt, 2021). *Code* represents classes and subclasses along a gradient of decreasing soil moisture.

Class	Soil class (WRB classification)	Subclass (German classification)	Codes
H	Histosol	HN Niedermoor (Fen)	100
		HH Hochmoor (Bog)	110
G	Gleysol	GN Nassgley	120
		GM Anmoorgley	120
		GH Moorgley	130
		GG Gley	140
S	Stagnosol	SH Haftpseudogley	160
		SG Stagnogley	170
		SS Pseudogley	180
A	Fluvisol	AB Vega	200
		AO Rambla	220
		AQ Paternia	220
		AZ Kalkpaternia	240
P	Podzols	PP Podzol	260
B	Cambisol	BB Pseudogley-Braunerde	280
		BB Braunerde	300
C	Terrae calcis	CF Terra fusca	320
		CR Terra rossa	330
L	Luvisol	LL Parabraunerde	340
		LF Fahlerde	NA
D	Pelosol	DD Pelosol	360
R	Leptosol (>10 cm topsoil)	RN Ranker	380
		RQ Regosol	400
		RR Rendzina	420
		RZ Pararendzina	440
O	Lithic Leptosol (<10 cm topsoil)	OO Syrosem	460
		OL Lockersyrosem (Unconsolidated Syrosem)	460
F	O/C Syrosem	FF Felshumusboden (Rock-Humus Soil)	460
		FS Skeletthumusboden (Skeletic Humus Soil)	460
Y	Anthropogenic soils	Kolluvisol, Hortisol, Rigosol, Treposol	NA
Z	Sealed, Water		NA

Table A3: Climatic and edaphic predictor variables included in the generalized boosted regression model for the occurrence of *Jacobaea aquatica* in Bavarian grasslands

Variable name	Meaning	Remark
rainMAM	Spring rainfall (March-May);	Data for 1988-1997 and 2008-2017 obtained from Deutscher Wetterdienst (opendata.dwd.de; downloaded on 10.1.2022), averaged and the original resolution of 1000 x 1000 m resampled to match the 50 x 50m resolution of the soil map. Future data for 2028-2037 under rcp4.5 and rcp8.5 scenario were obtained from Bayerisches Landesamt für Umwelt (downloaded 14.3.2022); original resolution 5000 x 5000 m resampled to match 50 x 50 m resolution of soil map.
rainJJA	Summer rainfall (June-August)	
rainSON	Autumn rainfall (September-November)	
airTempMin	All year minimum air temperature 2m above ground	
airTempMax	All year maximum air temperature 2m above ground	
IceDays	Number of days per year on which the air temperature does not go above 0°C	
FrostDays	Number of days per year on which the minimum air temperature is below 0°C	
HotDays	Number of days per year on which the maximum air temperature reaches or exceeds 30 °	
SoilClass	Soil class	Digital soil map was obtained as a shape file from the Bayerisches Landesamt für Umwelt 2021a; https://www.lfu.bayern.de/boden/karten_daten/uebk25/ (accessed 10.2.2022). Soil classes were recoded according to table A2 and converted into raster data with 50 x 50 m resolution.

Table A4: Tuning parameters for the gradient boosted model used to predict the habitat suitability for *Jacobaea aquatica* in Bavarian grasslands

Model parameter	Initial value	Tuned value
n.tree	5000	2500
Interaction.depth	7	7
n.minobsinnode	5	5
shrinkage	0.001	0.01
bag.fraction	0.5	0.5
train.fraction	0.5	0.7
cv.folds	3	3

Table A5: Assessment of the generalized regression models for the occurrence of *Jacobaea aquatica* in Bavarian grasslands related to soil type and climatic factors. PA1 … PA10: models for the respective set of pseudo absences; variable importance given as mean±SD; AVG averaged model

**Model performance**					**Relative variable importance**								
**Model**	**AUC**	**Cut off**	**Sensitivity**	**Specifity**	**rainMAM**	**rainJJA**	**rainSON**	**airTempMin**	**airTempMax**	**IceDays**	**FrostDays**	**HotDays**	**SoilClass**
PA1	0,962	0,168	93,8	88,9	4,6	49,7	1,8	5,8	8,6	3,9	6,0	2,9	16,7
PA2	0,946	0,364	87,6	88,9	5,2	55,2	1,9	6,9	3,7	7,8	6,3	1,5	11,6
PA3	0,975	0,512	86,4	98,8	3,6	49,2	2,0	5,8	7,5	5,5	7,7	2,7	15,9
PA4	0,966	0,465	91,1	98,5	5,1	49,3	1,3	5,0	5,0	7,8	5,1	3,0	18,3
PA5	0,948	0,171	92,6	86,4	7,3	51,9	3,1	8,1	5,8	4,0	5,0	2,1	12,9
PA6	0,953	0,263	91,4	90,1	4,6	49,7	1,8	5,8	8,7	3,9	6,0	2,9	16,7
PA7	0,941	0,642	86,4	93,8	6,2	47,8	2,6	7,1	5,5	6,5	6,5	2,8	15,2
PA8	0,965	0,288	92,9	95,9	5,1	49,3	1,2	5,0	5,0	7,8	5,1	3,0	18,3
PA9	0,972	0,309	92,4	93,8	14,8	37,8	4,8	6,8	6,4	4,3	8,0	2,0	16,0
PA10	0,971	0,288	92,9	97,0	7,3	51,9	3,1	8,1	5,8	4,0	5,0	2,1	12,9
**mean**	-	-	-	-	**6,4±3,2**	**49,2±4,5**	**2,4±1,1**	**6,4±1,1**	**6,2±1,6**	**5,4±1,8**	**6,1±1,1**	**2,5±0,5**	**15,5±2,3**
**AVG**	**0,961**	**0,350**	**91,4**	**97,7**	-	-	-	-	-	-	-	-	-

Table A6: Area at risk and risk changes on the distribution of *Jacobaea aquatica* in Bavarian grasslands within the respective periods

		Risk area in km²							
Time period	Climate change scenario	Total	High p > 80%	Moderate p 65-80%	Low p 50-65%	New	Not anymore	Increase *d*p > 20%	Decrease *d*p > 20%
1988-1997	-	23707	11052	2019	10636	-	-	-	-
2008-2017	-	11817	7979	2263	1575	1221	13692	1372	1438
2028-2037	Rcp4.5	9742	283	2633	6826	4582	4277	43	7385
2028-2037	Rcp8.5	5936	5	1028	4903	4544	3967	43	7693

p: probability of occurrence, *d*p: change in probability of occurrence; new, not anymore, increase and decrease: related to the previous period

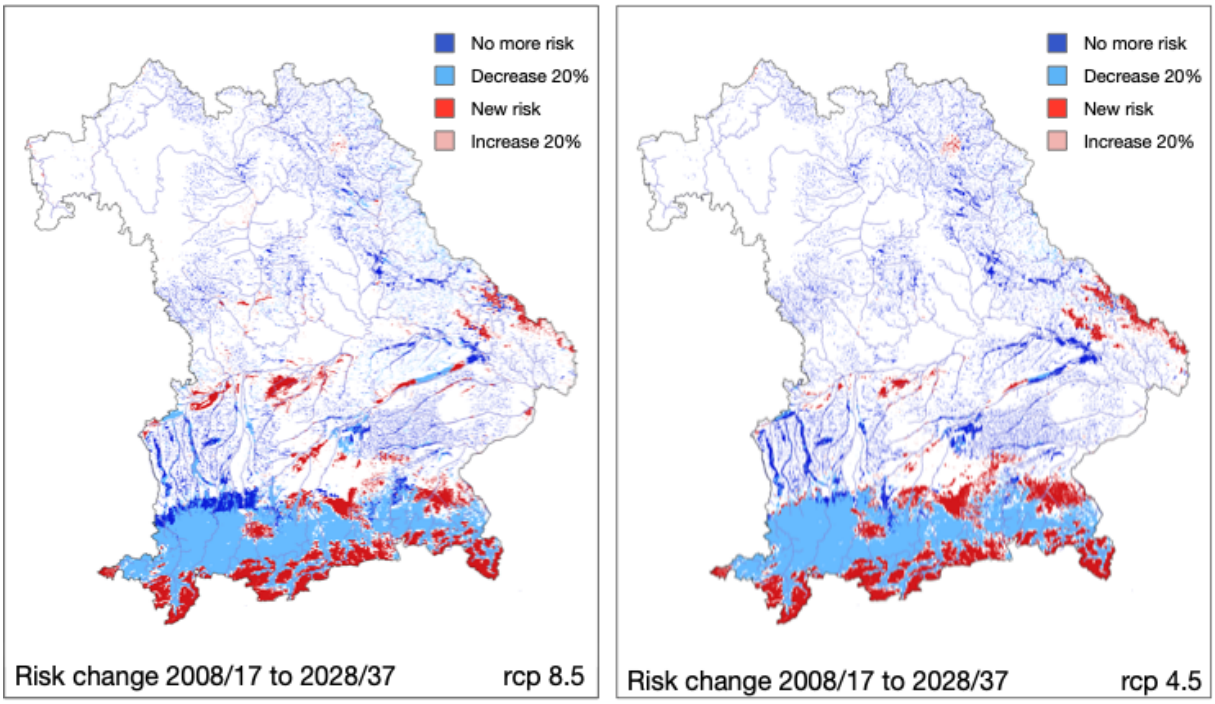

Figure A1: Risk change on the occurrence of *Jacobaea aquatica* in Bavarian grasslands as predicted by the gbm based on climatic factors and soil types for the future period 2028-2037 under rcp4.5 and rcp8.5 scenario.

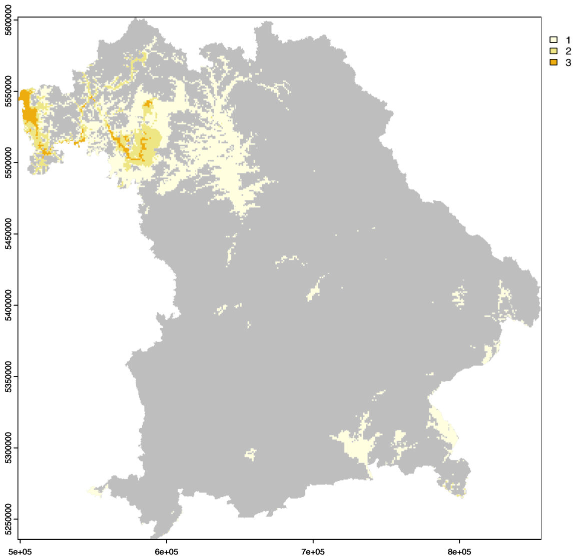

a) MOP: rcp45, all variables

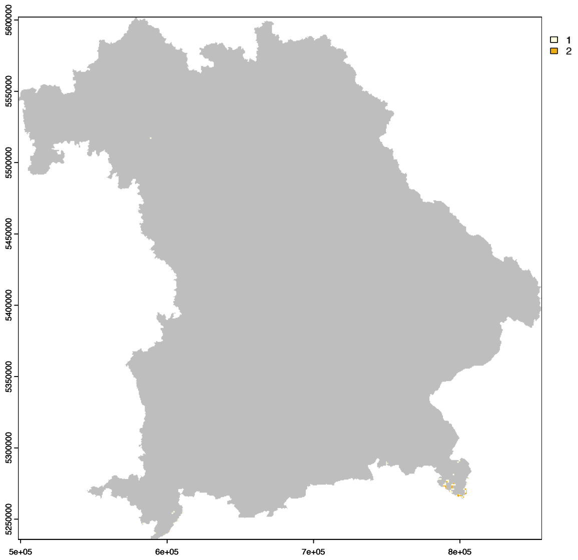

a) MOP: rcp45, without ice, frost and hot days

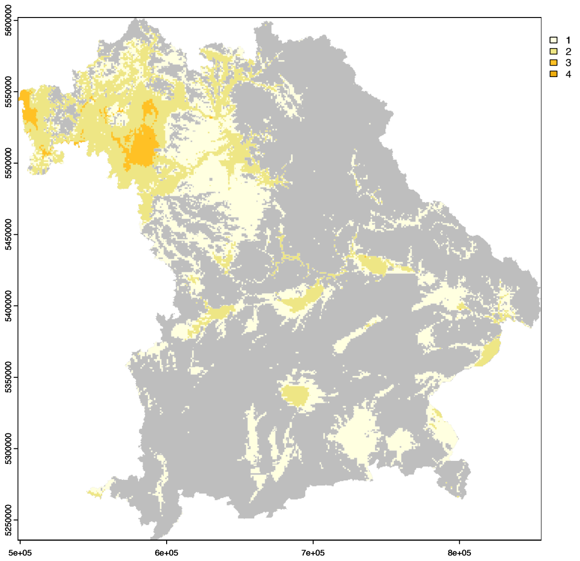

a) MOP: rcp85, all variables

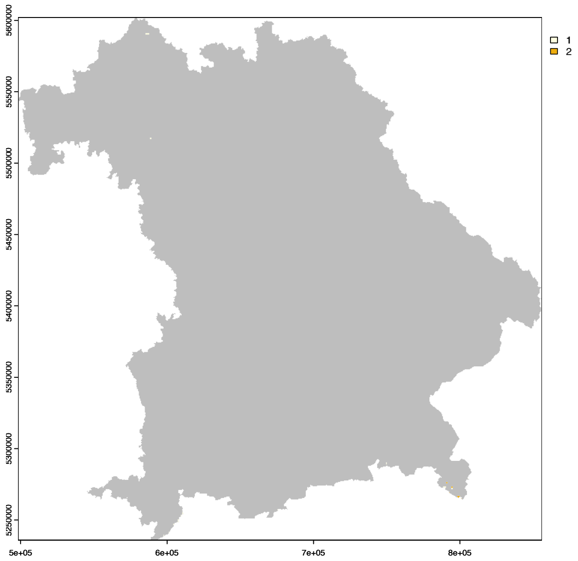

a) MOP: rcp85, without ice, frost and hot days

Figure A2: Maps of non-analogous cells in the rcp45 and rcp85 scenarios. a) Rcp45 scenario including all explanatory variables. (~11% non-analogous cells); b) Rcp45 scenario without including frost days, ice days and hot days (~0% non-analogous cells); c) Rcp85 scenario including all variables (~30% non-analogous cells); d) Rcp85 scenario without including frost days, ice days and hot days (~0% non-analogous cells).
